# Estimating chlorophyll content and photochemical yield of photosystem II (Φ_PSII_) using solar-induced chlorophyll fluorescence measurements at different growing stages of attached leaves

**DOI:** 10.1093/jxb/erv272

**Published:** 2015-06-12

**Authors:** Bayaer Tubuxin, Parinaz Rahimzadeh-Bajgiran, Yusaku Ginnan, Fumiki Hosoi, Kenji Omasa

**Affiliations:** ^1^Graduate School of Agricultural and Life Sciences, The University of Tokyo, 1-1-1 Yayoi, Bunkyo-ku, Tokyo 113–8657Japan; ^2^School of Forest Resources, The University of Maine, 5557 Nutting Hall, Orono, ME 04469, USA

**Keywords:** Chlorophyll content, different leaf age, Fraunhofer line depth principle, photochemical yield of photosystem II (ΦPSII), solar-induced chlorophyll fluorescence.

## Abstract

This paper illustrates the possibility of measuring chlorophyll (Chl) content and Chl fluorescence parameters by the solar-induced Chl fluorescence (SIF) method using the Fraunhofer line depth (FLD) principle, and compares the results with the standard measurement methods. A high-spectral resolution HR2000+ and an ordinary USB4000 spectrometer were used to measure leaf reflectance under solar and artificial light, respectively, to estimate Chl fluorescence. Using leaves of *Capsicum annuum* cv. ‘Sven’ (paprika), the relationships between the Chl content and the steady-state Chl fluorescence near oxygen absorption bands of O_2_B (686nm) and O_2_A (760nm), measured under artificial and solar light at different growing stages of leaves, were evaluated. The Chl fluorescence yields of ΦF 686nm/ΦF 760nm ratios obtained from both methods correlated well with the Chl content (steady-state solar light: R^2^ = 0.73; artificial light: R^2^ = 0.94). The SIF method was less accurate for Chl content estimation when Chl content was high. The steady-state solar-induced Chl fluorescence yield ratio correlated very well with the artificial-light-induced one (R^2^ = 0.84). A new methodology is then presented to estimate photochemical yield of photosystem II (Φ_PSII_) from the SIF measurements, which was verified against the standard Chl fluorescence measurement method (pulse-amplitude modulated method). The high coefficient of determination (R^2^ = 0.74) between the Φ_PSII_ of the two methods shows that photosynthesis process parameters can be successfully estimated using the presented methodology.

## Abstract (precis)

An estimation and comparison of chlorophyll content and photochemical yield of photosystem II obtained from solar and artificially induced chlorophyll fluorescence is presented here, along with a new technique to estimate photochemical yield from the solar-induced method.

## Introduction

Chlorophyll (Chl) fluorescence has been studied as a useful probe for photosynthesis research (Govindjee, 1995, Papageorgiou and Govindjee, 2004; Buschmann, 2007; Baker, 2008; Omasa *et al.*, 2009; van der Tol *et al.*, 2009; Kalaji *et al.*, 2012; Murchie and Lawson, 2013. It has been extensively applied for active phenotyping remote sensing of photosynthetic functioning, and for studying biotic and abiotic stress in plants (Omasa, 1998; Omasa *et al.*, 1987, 2007; Lichtenthaler *et al.*, 1992; Kolber *et al.*, 1998; Kim *et al.*, 2001; Omasa and Takayama, 2003; Schreiber, 2004; Moya *et al.*, 2004; Konishi *et al.*, 2009; Pieruschka *et al.*, 2012; 2014).

However, the common active remote sensing method might be limited to short-distance use for individual leaves and small plants. Efforts have been made to expand the application of active Chl fluorescence measurement methods for long-distance, canopy-scale studies using the laser induced fluorescence transient (LIFT) method. The LIFT approach employs low-intensity pulses instead of a saturating pulse to measure the ﬂuorescence transient, which is interpolated to a maximum ﬂuorescence level using a ﬂuorescence model. The technique is capable of measuring Chl fluorescence up to a distance of 50 m but there are still challenges for more long-distance remote sensing (Kolber *et al.* 2005; Pierurschka *et al.* 2012; 2014).

Passive remote sensing of steady-state solar-induced Chl fluorescence (SIF) has more recently become available (Meroni *et al.*, 2009). The advantages of the SIF method are that the technique does not require an artificial excitation source and it can be used on air-borne and space-borne devices for canopy and larger-scale applications. However, this technique requires instruments with high spectral resolution and high accuracy to be able to differentiate the weak fluorescence signal from the strong background light reflection (Meroni and Colombo, 2006).

SIF measurement techniques have been developed for field and air-borne applications in recent years (Plascyk *et al.*, 1975; Moya *et al.*, 2004; Liu *et al.*, 2005; Corp *et al.*, 2006; Meroni *et al.*, 2006, 2009; Rascher *et al.*, 2008; Liu and Cheng, 2010; Zarco-Tejada *et al.*, 2012). The development of space-borne SIF remote sensing of GOSAT satellite has provided the opportunity to assess global terrestrial carbon cycle and has attracted a considerable amount of attention within scientific communities (Frankenberg *et al.*, 2011; Joiner *et al.*, 2011; Porcar-Castel *et al.*, 2014).

Current trends of research in the SIF field demonstrate the need for further evaluations of this technique. There are intrinsic differences between the basis of active Chl fluorescence measurement methods based on pulse-amplitude modulated (PAM) measurement and that of the SIF; thus, consequent challenges are currently encountered. Further, leaf-level experimental work is needed to characterize relationships between SIF- and PAM-measured Chl fluorescence (Porcar-Castel *et al.*, 2014). The steady-state Chl fluorescence has been shown to be partially re-absorbed by Chl (Lichtenthaler and Rinderle, 1988; Buschmann, 2007), which necessitates the evaluation of Chl fluorescence at various life stages of the plants when Chl content is variable. In addition, interest in relationships between steady-state SIF and the photosynthesis reaction at the physiology level, as well as growth primary production and the enhanced vegetation index (Frankenberg *et al.*, 2011; Joiner *et al.*, 2011), which are related to biomass and Chl content, is growing (Van der tol *et al.*, 2014).

To progress our understanding of the potential to detect SIF signals from vegetation reflectance to study photosynthesis, the main objective of this paper is to evaluate the performance of the SIF measurement method throughout the lifespan of leaves with varying Chl content through comparison with the standard Chl measurement method. This is carried out via an estimation of Chl content and photochemical yield of photosystem II (Φ_PSII_). Not many studies have examined the correlation between both measurement methods of Chl fluorescence on the same sample. Moya *et al.* (2004) found high correlation between both ﬂuorescence measurements; however, the experiments were limited to a small range of Chl content (350–450mg m^−2^). As far as could be determined, there is currently no report available to estimate and compare large-range Chl content or Φ_PSII_ obtained from the two methods. In the present paper, the focus has, therefore, been on the relationships between the steady-state Chl fluorescence and Chl content near the oxygen absorption bands of O_2_B (686nm) and O_2_A (760nm), measured under artificial light and solar light at different growing stages of leaves. A new technique to estimate Φ_PSII_ from the SIF method is also presented.

## Materials and methods

### Plant material and growth conditions

Paprika (*Capsicum annuum* cv. ‘Sven’) plants were grown in an environmentally controlled growth chamber for 10–18 weeks. The plants were illuminated for 12h each day with fluorescent lights and halogen lamps at a photosynthetic photon flux (PPF) of 400 μmol m^–2^ s^–1^. The growth chamber air temperature was 25.0°C during the day and 20.0°C at night with relative humidity of ~70%. Plants were watered daily with a nutrient solution (1:1000 dilution of HYPONeX). Fully expanded mature and aging leaves of different growth stages were used in the experiments.

### Chl fluorescence measurement under artificial light

Comparable veinless sites of attached leaves were used to measure the steady-state artificial-light-induced Chl fluorescence. The fluorescence spectra of leaves were measured at an interval of 500ms (integration time) with a USB4000 spectrometer (Ocean Optics, USA) under a halogen light (Sumita Optical Glass, Inc., LS-100F) through short-pass (Optical Coatings Japan, wavelength λ < 600nm) and heat-absorbing filters in a dark room. The USB4000 spectrometer was set to the 350–850nm range with 3648 channels, grating of 600 lines mm^−1^, and a 50 μm slit. It was connected to a 3 m-long fibre cosine corrector-diffuser. The instrument has 1.5–2.3nm full width at half maximum (FWHM). The attached leaf was set at a 45° angle to the optical light beam and the fibre optic diffuser was set at 5cm distance from the leaf surface. The fibre-optic diffuser was equipped with a long-pass filter (Optical Coatings Japan, λ > 640nm) to block the reflected light from the leaf. The LS-1-CAL calibrated tungsten halogen NIST-traceable light source (Ocean Optics, USA) was used to calibrate the spectrometer. The leaf area was illuminated for 20min (PPF: 150 μmol m^–2^ s^–1^), and then the steady-state Chl fluorescence was measured. Black wool paper was set at the back of the leaf to remove the reflection of light transmitted through the leaf. Chl fluorescence spectra were recorded to computer via a USB port using SpectraSuite operating software.

### Spectral radiant intensity measurement for chlorophyll content estimation under solar light using the FLD method

After the above-mentioned artificial-light-induced measurements, the spectral radiant intensity of the same leaf area was measured at an interval of 200ms (integration time) under solar light using a 0.035nm FWHM HR2000+ spectrometer (Ocean Optics, USA) in the 680–770nm range with 2048 channels. The spectrometer was built with grating H11 of 1800 lines/mm, a 5 μm slit, an L2 detector collection lens, an OF1-OG590 long-pass filter, and a set of high-reflectivity AgPlus mirrors model SAG+UPGD-HR. The spectrometer was connected to a 2 m-long fibre optic, bundled 600 μm optical fibre up, with a CC-3 VIS-NIR cosine corrector-diffuser for spectral radiant intensity measurement. The attached leaf was set vertically to incident solar light after sufficient light adaptation. The fibre-optic diffuser was set at 5cm distance from the leaf surface, and it was held at 45° to light to reduce specular reflection from the leaf surface. An 18% Gray Card (Kodak, USA) set at the same angle and position as the leaf was used as the non-fluorescent reference standard. Both the spectra of the leaf and the non-fluorescent reference were recorded to computer via a USB port using OPwave+ operating software (Ocean Optics, USA). Black wool paper was set at the back of the leaf to remove the reflection of light transmitted through the leaf. During the experiments, a LI-COR LI-250 light meter was used to measure the PPF (800–1800 μmol m^–2^ s^–1^).

### Spectral radiant intensity measurement for Φ_PSII_ estimation under solar light

The attached leaf was set vertically to incident solar light using a JUNIOR-B leaf clip (Walz). The fibre-optic diffuser of the HR2000+ spectrometer was set at 5mm distance from the leaf surface, and it was held at 45° to solar light. During the experiment, the spectral leaf radiant intensity was continuously measured at an interval of 200ms (integration time) under solar light using the spectrometer after sufficient light adaptation. The saturation light pulse of about 5000 μmol m^–2^ s^–1^ from a red laser (KaLaser: 660nm, 200 mW) was illuminated for 1 s at 60° to the leaf from a distance of 40cm and at about a 30mm diameter footprint. For the verification, the photochemical yield of photosystem II (Φ_PSII_) was measured at a 1mm distance from the leaf by JUNIOR PAM (about 10000 μmol m^–2^ s^–1^ blue LED saturation pulse for 0.6 s and measuring beam of 5 Hz) to give Φ_PSII, PAM_. The spectral radiant intensity data were recorded by the above-mentioned computer system. The set of sequential experiments included (i) measurement of solar light radiant intensity reflected from a non-fluorescent reference (90% White Card), (ii) measurement of solar light radiant intensity from the attached leaf, (iii) measurement of saturation laser pulse radiant intensity from the leaf, (iv) measurement of Φ_PSII, PAM_ of the leaf using JUNIOR PAM, (v) measurement of solar light radiant intensity from the leaf, (vi) measurement of the second laser pulse radiant intensity from the leaf, and (vii) measurement of solar light radiant intensity reflected from the non-fluorescent reference at each interval of 20 s in (ii) to (vi) and about 60 s in (i) and (vii) under solar light. A data set with very little change in solar light intensity during the experiment was used for the calculation of Φ_PSII_ estimated by the Fraunhofer line depth principle (FLD), designated Φ_PSII, FLD._ The solar light intensity in the experiment was 200–1600 μmol m^−2^ s^−1^.

### Solar-induced Chl fluorescence estimation using FLD

It is difficult to quantify the SIF from the ordinary spectral leaf radiant intensity because the signal is obscured by the reflected light. However, the leaf radiant intensity in Fraunhofer lines increases the ratio of Chl fluorescence emission to reflected light in the leaf radiant intensity as compared with other wavelengths, hence the fluorescence emission can be quantified by the FLD (Lichtenthaler *et al.*, 1992; Moya *et al.*, 2004).

The FLD method was originally proposed by Plasccyk and Gabriel (1975), then successfully used by Meroni *et al.* (2006; 2008*a*) and Rascher *et al.* (2009) at the leaf level, and at a canopy level by Moya *et al.* (2004), Meroni *et al.* (2008*b*), and Daumard *et al.* (2012). At the ground level, two oxygen absorption bands of terrestrial atmosphere at 760nm (O_2_A) and 686nm (O_2_B) are usually used to estimate the steady-state SIF because the absorption bands include Chl fluorescence emission spectra.

**Fig. 1.**
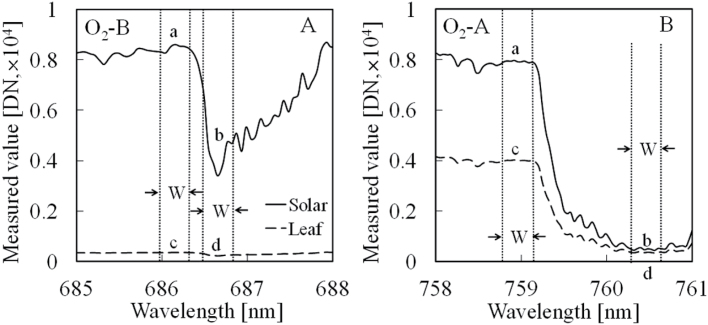
Concept of Fraunhofer line depth (FLD) method. (A) O_2_B band. (B) O_2_A band. The solid line is non-fluorescent reference radiant intensity spectra and the dashed line is paprika (*Capsicum annuum* cv. ‘Sven’) leaf radiant intensity spectra measured in O_2_A and O_2_B bands. The bands *a* and *c* are the border of the well and the bands *b* and *d* are the bottom of the well for the reflectance and leaf. The W is wavelength width in band a, b, c, and d. The measured value of radiant intensity is shown as the digital number (DN) and is not calibrated to exact radiance. The leaf Chl content was 621.1mg m^−2^.

Fig. 1 shows the FLD method. In Fig. 1A, B, the solid line is the non-fluorescent reference radiant intensity spectra and the dashed line is the leaf radiant intensity spectra measured in O_2_A and O_2_B bands. M_a_ and M_b_ are the mean values of radiant intensity (digital numbers, which is not calibrated to exact radiance) from the non-fluorescent reference in band a (border of the well, A: W = 685.93 to 686.32nm; B: W = 758.76 to 759.17nm; W: the wavelength width) and in band b (bottom of the well, A: W = 686.54 to 686.93nm; B: W = 760.24 to 760.64nm), and M_c_ and M_d_ are the mean values of radiant intensity from the leaf in band c (the same W as a) and in band d (the same W as b), respectively. Following Plascyk and Gabriel (1975) and Moya *et al.* (2004), the reflectance coefficient (R) and the solar-induced fluorescence intensity (Fs) were derived from the measured values of M_a_, M_b_, M_c_ and M_d_ as follows:

$$\text{R}=\left({\text{M}}_{c}-{\text{M}}_{d}\right)/\left({\text{M}}_{a}-{\text{M}}_{b}\right)$$ (1)

$$\text{Fs}={\text{M}}_{d}–\text{R}\times {\text{M}}_{b}$$ (2)

When the saturation laser pulse-induced fluorescence intensity under solar light is calculated by equations (1) and (2), the radiant intensity M_a_ and M_b_ from the non-fluorescent reference under only solar light are used because the radiant intensity in a and b bands of O_2_A is not affected by the red laser pulse. The radiant intensity M_c_ and M_d_ apply to those from the leaf under both solar and laser lights.

### Chl fluorescence yield ratio

The Chl fluorescence yield (ΦF, the ratio of fluorescence photons to absorbed photons) can more accurately represent the relationship between the Chl fluorescence signal magnitude and light intensity than Chl fluorescence intensity (Govindjee, 1995). The solar-induced and artificial-light-induced Chl fluorescence yields near the 686nm and 760nm bands were approximately calculated by

$$\Phi {\text{F}}_{s}=\text{F}/\left(\text{PPF }\times \;0.\text{84}\right)$$ (3)

where F is the fluorescence intensity (relative units), PPF is the photosynthetic photon flux (μmol m^–2^ s^–1^), and 0.84 is the leaf absorption coefficient. The solar-induced (ΦF_s_) and artificial-light-induced (ΦF_a_) Chl fluorescence yield ratios of ΦF_s_686.7/ΦF_s_760.4 and ΦF_a_686.4/ΦF_a_760.5 were calculated and their relationships at different Chl content levels compared. The ratio of [ΦF_s_686.7/ΦF_s_760.4]/[ΦF_a_686.4/ΦF_a_760.5] was then calculated to assess the steady-state SIF at different leaf-growing stages. The wavelength width (W in Fig. 1) to minimize the mean absolute error of SIF estimation was 0.4nm.

### Φ_PSII, FLD_ estimation from solar-induced and red laser saturation pulse-induced Chl fluorescence yields

From a set of temporal changes in the above-mentioned spectral radiant intensity measurement of (1) to (7), the changes in the SIF yield (ΦF760.4) and laser saturation pulse-induced yield (ΦFm’760.4) of the O_2_A band were calculated by the FLD method. Φ_PSII, FLD_ was calculated by

$$\Phi_{\text{PSII},\text{ FLD}}=(\Phi \text{Fm}’-\Phi \text{Fs})/\Phi \text{Fm}’$$ (4)

where ΦFs is a constant value after median filtering to data measured during the 10 s just before the saturation laser pulse for noise reduction, and ΦFm’ is the mean value of the highest three data points during the saturation laser pulse.

### Leaf spectral absorptance

Chl fluorescence spectra largely overlap with Chl absorption, and the red fluorescence re-absorbed by Chl itself is larger than the far-red fluorescence (Govindjee, 1995; Gitelson *et al.* 1998). To investigate the re-absorption in Chl fluorescence spectra, the reflectance (R) and transmittance (T) of the leaf were measured using a spectrophotometer equipped with an integrating sphere (V570, JASCO, Japan) in a range of 600–800nm and a spectral resolution of 2nm. The reflectance spectra were measured against barium sulphate as a reference standard; black wool paper was set at the back of the leaf to remove the re-reflection of light transmitted through the leaf. Leaf spectral absorptance was calculated as 1-T-R (Buschmann *et al.*, 1994; Gitelson *et al.*, 1998, 2003).

### Chlorophyll content

A 1.5 cm-diameter leaf disc was cut from the marked point of the leaf using a standard leaf punch. After weighing, the leaf disc was mashed in 96% ethanol solution using a mortar and a pestle. The pigments were completely separated in sedimentation tubes by a centrifugal separator set for 5min at 2000rpm. The absorption spectrum of the top layer solution in the tube was measured by a V570 spectrophotometer (JASCO, Japan) at 0.5nm intervals between 600nm and 800nm. Total Chl content was calculated from the absorption spectra using equations reported by Wintermans and de Mots (1965).

## Results

**Fig. 2.**
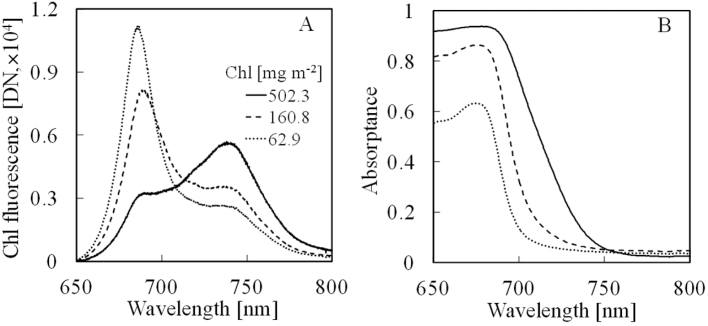
Steady-state Chl fluorescence emission spectra and absorption spectra of different Chl content leaves taken under artificial illumination (λ < 600nm). (A) The steady-state Chl fluorescence emission spectra. (B) Absorptance spectra of the same leaf sample. Chl content: the solid line is 502.3mg m^−2^, the dashed line is 160.8mg m^−2^, and the dotted line is 62.9mg m^−2^.

Figure 2A shows the steady-state Chl fluorescence emission spectrum of three leaves with different Chl content taken under the artificial illumination light (λ < 600nm). Two maxima appeared in the red region around 686nm (F686) and far-red region around 740nm (F740). Near the red region, Chl fluorescence increased sharply with the reduction of Chl content, but near the far-red region, it increased with the increase of Chl content. The Chl absorptance near 686nm greatly increased with increasing Chl content, whereas there was little difference near 760nm (Fig. 2B).

**Fig. 3.**
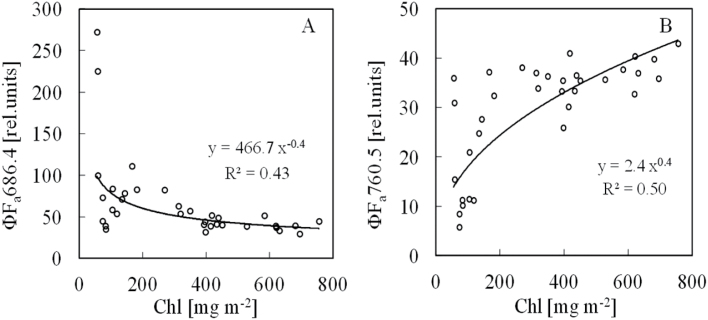
Relationships between leaf Chl content and steady-state artificial-light-induced Chl fluorescence yields. (A) ΦF_a_686.4. (B) ΦF_a_760.5.

Figure 3 shows relationships between leaf Chl content and steady-state Chl fluorescence yields of ΦF_a_686.4 and ΦF_a_760.5 under artificial light. The Chl fluorescence yields of ΦF_a_686.4 decreased with an increase in Chl content (Fig. 3A), whereas those of ΦF_a_760.5 increased (Fig. 3B), although the yields greatly varied at low Chl content below about 200mg m^−2^. At higher Chl content with more than about 400mg m^−2^, the changes in ΦF_a_686.4 and ΦF_a_760.5 become small.

**Fig. 4.**
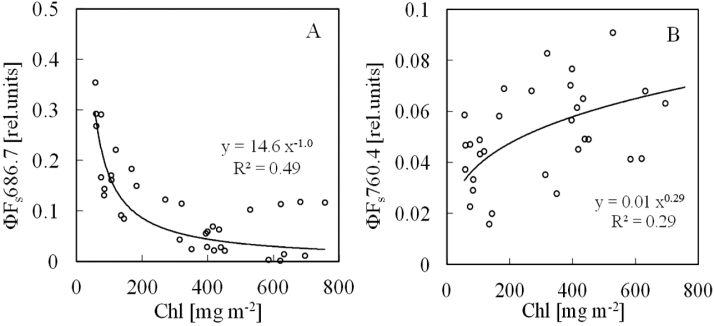
Relationships between leaf Chl content and steady-state SIF yields. (A) ΦF_a_686.7. (B) ΦF_a_760.4.

Figure 4 shows the relationships between leaf Chl content and steady-state solar-induced Chl fluorescence yields of ΦF_s_686.7 and ΦF_s_760.4. The Chl fluorescence yields of ΦF_s_686.7 decreased with an increase in Chl content (Fig. 4A), whereas the Chl fluorescence yields of ΦF_s_760.4 increased, although the yields varied largely, especially in ΦF_s_760.4 (Fig. 4B). Comparing Figs. 3 and 4, the solar-induced Chl fluorescence yield variation was larger than that induced by artificial light, probably because of a weak Chl fluorescence signal, lack of the sensitivity of the spectrometer, and solar fluctuation during the measurement.

**Fig. 5.**
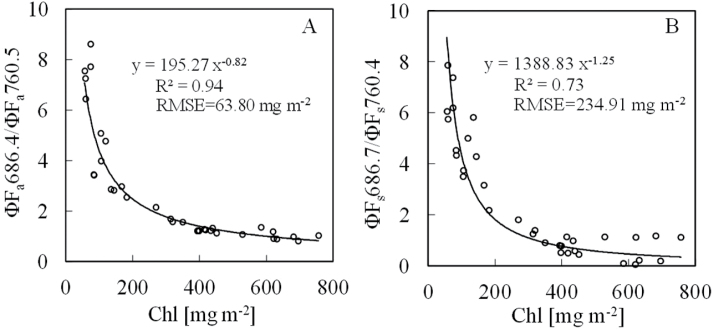
Relationships between leaf Chl content and steady-state Chl fluorescence yield ratios calculated from Figs. 3 and 4, respectively. (A) Artificial-light-induced Chl fluorescence yield ratio (ΦF_a_686.4/ΦF_a_760.5). (B) SIF yield ratio (ΦF_s_686.7/ΦF_s_760.4).

Figure 5 shows the relationships between leaf Chl content and steady-state Chl fluorescence yield ratios calculated from Figs. 3 and 4. Under artificial light, the ratio of ΦF_a_686.4/ΦF_a_760.5 showed a very clearly inverse curvilinear relationship with the Chl content of leaves (Fig. 5A; R^2^ = 0.94). A similar highly inverse curvilinear relationship was also obtained under solar light (Fig. 5B; R^2^ = 0.73) regardless of the large variation shown in Fig. 4. The lower coefficient of determination value obtained for the solar light-induced yield ratio is caused by wider variation at all Chl content values. A comparison of root mean square error (RMSE) values as indicated on the figure shows a much larger RMSE for the SIF method. However, when RMSEs are calculated for Chl content values below 400mg m^−2^, the RMSEs of the two methods become closer and amount to a maximum of 40mg m^−2^ for the SIF method, indicating that the SIF method might be less accurate for higher Chl content (above 400mg m^−2^) estimation.

**Fig. 6.**
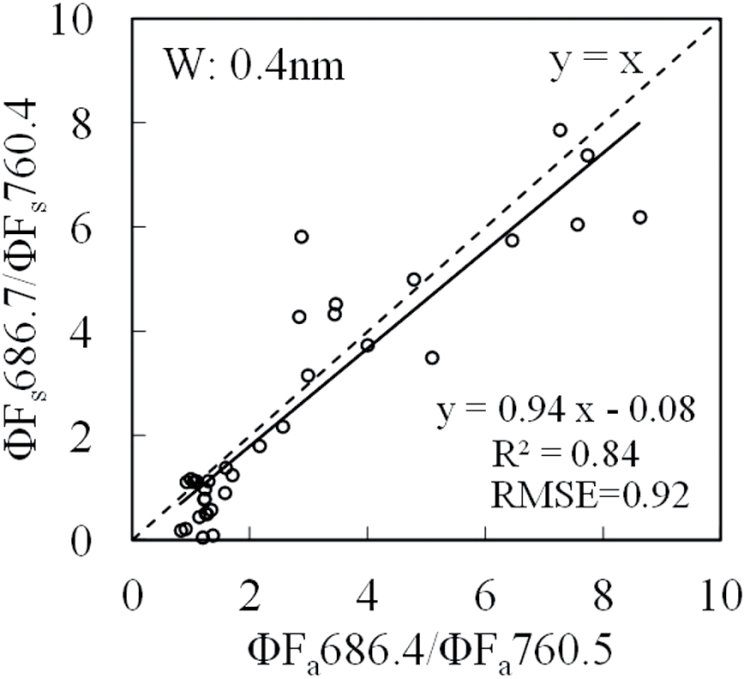
Relationship between artificial-light-induced Chl fluorescence yield ratio (ΦF_a_686.4/ΦF_a_760.5) and solar-induced yield ratio (ΦF_s_686.7/ΦF_s_760.4) at different growing stages of paprika leaves.

Figure 6 shows a linear correlation between artificial-light-induced Chl fluorescence yield ratio (ΦF_a_686.4/ΦF_a_760.5) and the solar-induced one (ΦF_s_686.7/ΦF_s_760.4) obtained at the optimal W (W = 0.4nm). The regression line was very close to the y = x line.

**Fig. 7.**
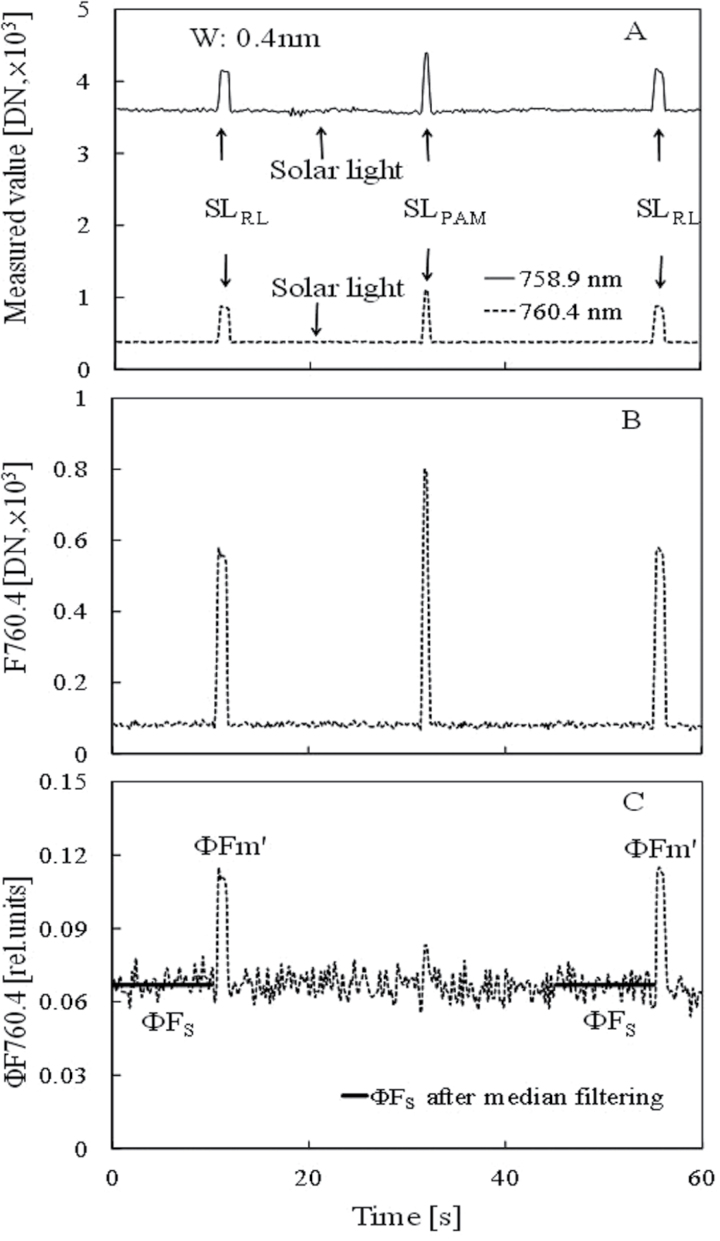
Measurement of Chl fluorescence using the FLD method under solar light and saturation pulse for estimating Φ_PSII_ of paprika leaf. (A) Temporal changes in leaf radiant intensity spectra measured using a HR2000+ spectrometer under solar light (1450 μmol m^−2^ s^−1^), where SL_RL_ is the saturation light pulse (4450 μmol m^−2^ s^−1^) using red laser (660nm) and SL_PAM_ is the saturation light pulse (blue LED, about 10 000 μmol m^−2^ s^−1^) of JUNIOR PAM. The solid line is the spectra of 758.9nm and the dotted line is that of 760.4nm. A mean value of 0.4nm wavelength width (W) was used. (B) Temporal changes in Chl fluorescence intensity (F760.4) calculated by the FLD method. (C) Temporal changes in Chl fluorescence yield (ΦF760.4). The dotted line is the changes in ΦF760.4 and the solid line is the value after median filtering during the 10 s before the SL_RL_ illumination for estimating Φ_PSII_.

Figure 7 shows an example of Chl fluorescence measurement using the FLD method under solar light and saturation pulse for estimating Φ_PSII_ of the attached leaf. The temporal changes in radiant intensity measurement of (ii) to (vi) mentioned in Materials and methods, Spectral radiant intensity measurement for Φ_PSII_ estimation under solar light, are shown in Fig. 7A. The radiant intensity increased with the saturation pulses of 660nm laser (SL_RL_) and blue LED (SL_PAM_) of JUNIOR PAM. Fig. 7B, C shows temporal changes in Chl fluorescence intensity (F760.4) calculated by the FLD method and those in Chl fluorescence yield (ΦF760.4) calculated by equation (3). The solid line in Fig. 7C is the sunlight fluorescence yield (ΦF) without noise after median filtering during 10 s before the SL_RL_ illumination for estimating Φ_PSII_. The saturation laser-induced fluorescence yield (ΦFm’) under solar light was larger than the yield induced by SL_PAM_. This result might have been caused by the difference in beam footprint sizes between the HR2000+ spectrometer and JUNIOR PAM.

**Fig. 8.**
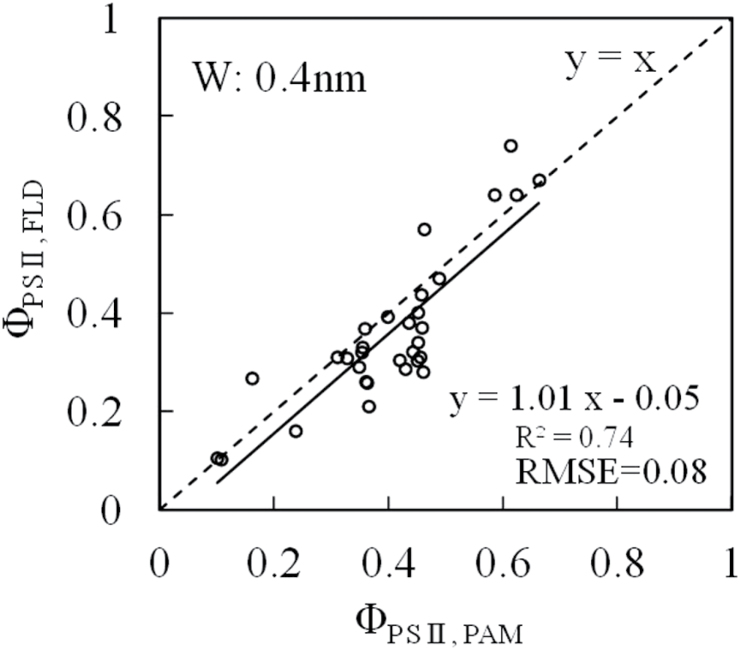
Relationship between Φ_PSII, PAM_ and Φ_PSII, FLD_ at different growing stages of paprika leaves. The SL_RL_ was about 5000 μmol m^−2^ s^−1^ and the solar light intensity was 200–1600 μmol m^−2^ s^−1^.

Figure 8 shows a linear correlation between Φ_PSII, PAM_ (measured by JUNIOR PAM) and Φ_PSII, FLD_ (estimated by FLD method) at different growing stages of the attached leaves. The regression line was approximated by y = x.

## Discussion

Active Chl fluorescence analysis such as the PAM method to assess photosynthesis activities is the most common method employed for laboratory-scale photosynthesis studies and can provide invaluable information on the factors affecting the photosynthesis process. However, this method has limitations for large-size plants or at canopy level. For this reason, the passive remote sensing of photosynthesis can be more feasibly carried out either through SIF studies or the photochemical reflectance index (PRI) measurement introduced by Gamon *et al.* (1990). Although PRI has been widely used for photosynthesis measurement, recent studies have shown that PRI use may be limited by its sensitivity to plant pigment variation at various life stages of the leaves (Rahimzadeh-Bajgiran *et al.*, 2012).

A few studies have been recently performed on the solar-induced Chl fluorescence at both laboratory scale and canopy level for plant photosynthesis measurement (Moya *et al.* 2004; Meroni *et al.*, 2006; 2009; Rascher *et al.*, 2008). Under solar light, the steady-state Chl fluorescence cannot be measured directly, because the photon emission from the canopy is less than 1% of what is emitted by the sun (Liu *et al.*, 2005). Hence, the SIF measurement using Fraunhofer line spectra such as the FLD principle has been proposed as the only way to remotely sense Chl fluorescence in natural environments. As outlined by others (Malenovský *et al.*, 2009; Porcar-Castel *et al.*, 2014; Van der Tol *et al.*, 2014), however, there is a need for further studies relating the steady-state SIF measurements to photosynthetic performance of plants.

The result presented here show that the steady-state Chl fluorescence emission spectrum in the red peak near 686nm strongly overlaps with the maximum of the leaf absorption spectrum near 680nm (Fig. 2). At the red region near O_2_B (686nm), the steady-state Chl fluorescence yield decreased sharply with the increase of Chl content regardless of the type of the light source-artificial or solar (Figs. 3A and 4A). This is speculated to be due to the fact that a large part of the red Chl fluorescence is re-absorbed by the Chl pigment before it can be measured. At the far-red region near O_2_B (760nm), because of the sharp decrease of the absorption of the leaf, the Chl fluorescence yield rose with Chl content increase (Figs. 3B and 4B). The phenomenon that Chl fluorescence is strongly re-absorbed by Chl pigment near the red region more than the far-red region has previously been reported (Lichtenthaler *et al.*, 1986; Gitelson *et al.*, 1998; Buschmann, 2007). The present results obtained by the FLD principle using O_2_A and O_2_B bands followed the same phenomenon.

Under solar light, the Chl fluorescence signal largely overlapped with the high-reflection signal of incident solar light and the re-absorption bands of pigments, and it is easily affected by the instrument conditions, the intensity of exciting light, and the angle of detection. The measurement of O_2_A and O_2_B reflectance under solar light is very difficult and one should be careful to provide identical illumination to the sample and the reference, because the depth of oxygen absorption bands may change easily (Zarco-Tejada *et al.*, 2003; Moya *et al.*, 2004; Meroni *et al.*, 2006; Campbell *et al.*, 2008). In this study, the reflectance of the standard reflectance panel was measured immediately after the measurement of the sample reflectance, but changes in the oxygen absorption bands leading to measurement errors are inherently inevitable. Also, under the unstable solar light intensity, the instability of photosynthetic states results in a change in the percentage of Chl fluorescence emission and Chl re-absorption capacity (Buschmann, 2007).

In practice, the Chl fluorescence intensity ratio of F685/F730, F690/F735, or F690/F740 is used to assess the steady-state artificial-light-induced Chl fluorescence signals and determine the Chl content in a non-destructive way (Agati *et al.* 1995; Buschmann, 2007; Campbell *et al.*, 2008). Gitelson *et al.* (1998) pointed out that ≥90% of the variation in the fluorescence ratio F690/F735 is exclusively dominated by Chl content. In contrast, at the ground level, two oxygen absorption bands of terrestrial atmosphere at 686nm (O_2_B) and 760nm (O_2_A) are usually used to estimate steady-state SIF because the position of the absorption bands are very close to the position of the Chl fluorescence spectrum peaks (Moya *et al.*, 2004; Meroni *et al.*, 2009). Here, the Chl fluorescence yield ratio of O_2_A and O_2_B was used to estimate the change of steady-state Chl fluorescence with Chl content and was considered to better express the relationship between the Chl fluorescence and illumination light.

The steady-state artificial-light-induced Chl fluorescence yield ratio of ΦF_a_686.4/ΦF_a_760.5 and the steady-state SIF yield ratio of ΦF_s_686.7/ΦF_s_760.4 showed very clear inverse curvilinear correlations with Chl content with R^2^ values of 0.94 and 0.73, respectively (Fig. 5). Moya *et al.* (1992) mentioned that a strong inverse relationship existed between fluorescence quantum yield and Chl content. Owing to the strong re-absorption influence of Chl pigment for Chl fluorescence near the 686nm band, the Chl fluorescence yield ratios of ΦF_a_686.4/ΦF_a_760.5 and ΦF_s_686.7 /ΦF_s_760.4 decreased with the increase of Chl content. Similar trends and shapes of the curves can be observed in Fig. 5A, B. However, the coefficients of regression lines are different. This is solely related to the lower accuracy of either measurement method to estimate Chl content at higher Chl content values. Up to a Chl content of about 300mg m^−2^, the coefficients of the power law functions are very similar with close R^2^ values (y = 223.2x^−0.851^, R^2^ = 0.80 for Fig. 5A; y = 241.4x^−0.867^, R^2^ = 0.79 for Fig. 5B). Once data pertaining to higher Chl content are included, the curves start to divert from each other, causing considerable differences in coefficients. This shows that both the active and passive methods used here have the same limitation at higher Chl content: under solar light, when the Chl content is very high (more than 400mg m^−2^), the value of ΦF_s_686.7/ΦF_s_760.4 becomes disperse; it appears that sometimes the Chl fluorescence yield ratio is difficult to estimate because of the too-low fluorescence intensity due to the high re-absorption of Chl pigment (Fig. 5B).

At the band width of 0.4nm, the steady-state Chl fluorescence yield ratios of the two methods were very well correlated with an R^2^ of 0.84 (Fig. 6). The work by Moya *et al.* (2004) reported a high correlation between active and passive fluorescence measurements (R^2^ = 0.99) (not the ratio, just absolute values), but this was obtained over a limited range of Chl content, eliminating the effects of re-absorption.

The saturation pulse method has been proposed as a powerful tool for assessing photosynthetic parameters (Genty *et al.*, 1989; Bilger and Björkman, 1990; Schreiber 2004) and many useful Chl fluorescence parameters, such as Φ_PSII_ and non-photochemical quenching, have been developed and used as indices of photosynthetic activity under solar and artificial (actinic) lights. The value obtained by multiplying the PPF value of actinic light by the Ф_PSII_ value is used as an indicator of photosynthetic electron transport rate and CO_2_ fixation rate (Baker and Oxborough, 2004; Schreiber, 2004).

The PAM Chl fluorometer is the standard instrument for the measurement of the Chl fluorescence parameters using the saturation pulse method. Therefore, the Chl fluorescence parameter Φ_PSII,FLD_ estimated using the FLD method under solar light and saturation pulse was verified with a PAM Chl fluorometer (JUNIOR PAM) according to the sequence shown in Fig. 7. Although the Chl fluorescence signal estimated by the FLD method was very noisy, the stable ΦFs760.4 and ΦFm’760.4 in equation (4) for calculating Φ_PSII, FLD_ were obtained as a constant value by the median filtering from data measured during the 10 s just before the saturation laser pulse (SL_RL_) and averaging of the highest three data points during the saturation laser pulse. To assure the reliability in Φ_PSII, FLD_ estimation, the small change in solar intensity was confirmed during a sequence from (i) to (vii) in the measurement mentioned in the Materials and methods. As a result, the ΦFs760.4 and ΦFm’760.4 values were stable during double measurements by the saturation laser pulse. Though the JUNIOR PAM blue LED saturation pulse (SL_PAM_) during 0.6 s was illuminated on the leaf at about 10000 μmol m^–2^ s^–1^, the Chl fluorescence yield estimated by the FLD method hardly changed (Fig. 7C). This could be owing to the measured error of SL_PAM_ (Fig. 7A), caused by the difference in beam footprint size between the HR2000+ spectrometer and JUNIOR PAM.

The Φ_PSII, FLD_ and Φ_PSII, PAM_ exhibited a good correlation with R^2^ of 0.74 and RMSE of 0.08, regardless of different leaf ages and solar intensities of 200–1600 μmol m^−2^ s^−1^ (Fig. 8). The regression line was almost y = x. This result is therefore introduced as a newly developed Φ_PSII_ measurement technique using the FLD method. Potential sources of error may include noise in the spectrometer, the solar fluctuation during the measurement, differences in the angle of the uneven leaf surface and non-fluorescent reference, the accuracy of light intensity measurement on the leaf, etc. These should be taken into consideration when trying to develop specific instruments designed based on the findings presented here.

Should the remote sensing of Φ_PSII_ for whole-plant and canopy scale studies using the SIF method be desired, a high-power laser or LED for the saturation pulse would be needed. This is a limitation of the saturation pulse method, but using the special sequence of laser illumination employing low-intensity pulses as described in Kolber *et al.* (1998), it will be possible to interpolate to a maximum ﬂuorescence level. Therefore, there is the possibility for remote sensing of Chl fluorescence parameters such as Φ_PSII_ without the use of accurate pulse-synchronized and modulated fluorimetric techniques such as PAM and LIFT using the SIF method and the illumination technique together.

Based on what was discussed above, the SIF measurement method could be used for both the Chl content estimation and photosynthesis parameter measurement (Φ_PSII_) needed for phenotyping studies of plants and remote sensing at different scales. In general, the SIF (FLD) method needs a high-spectral resolution and a high-sensitivity low-noise spectrometer to perform well. With more developments in instrument design and capability expected, the SIF method could potentially work well and perform more effectively should more research be focused on the development of spectrometers. For instance, if a cooled high-spectral resolution spectrometer with high sensitivity and low noise is used, more stable and low-noise data could be obtained.

## Conclusion

This paper illustrates the possibility of measuring Chl content and Chl fluorescence parameters using SIF measurement (FLD method) and the saturation pulse method via a red laser at different life stages of plant leaves. The results were compared with those obtained through the standard Chl fluorescence measurement method. A high-resolution HR2000+ spectrometer was used to measure leaf reflectance under solar light using the FLD method to estimate the solar-induced Chl fluorescence. A USB4000 spectrometer measured the steady-state artificially induced Chl fluorescence spectrum in laboratory conditions.

The steady-state SIF yield ratio of O_2_A and O_2_B showed high correlation with Chl content but the relationship was weaker when comparing with results obtained using artificial light. Both methods proved to be less accurate at higher Chl content owing to the strong re-absorption influence of Chl pigment for Chl fluorescence, with the SIF method being less accurate than the artificial-light-induced method. The steady-state SIF yield ratio and the steady-state artificial-light-induced Chl fluorescence yield ratio were very well correlated (R^2^ = 0.84), indicating that Chl content can be estimated from the proposed SIF measurement method.

A methodology was presented to estimate Φ_PSII_ from the SIF measurements, and verified against the active Chl fluorescence method (PAM). The high coefficient of determination found between Φ_PSII_ of the two methods shows that photosynthesis process parameters can be successfully estimated using the presented methodology. This can be the basis for future developments in remote sensing technologies for the estimation of both Chl content and Chl fluorescence parameters for passive phenotyping.

To scale-up the measurement techniques using SIF, measures should also be taken to remove the effect of noise, such as high background radiation, terrestrial atmosphere influence, and instrument measurement errors. Further studies are also recommended to evaluate these relationships beyond single leaf studies toward heterogeneous canopies.

## Abbreviations:

ΦF band: Chl fluorescence yield at O_2_A or O_2_B band (nm), where the subscript means the same as that in F
ΦFm’ band: laser saturation pulse-induced Chl fluorescence yield at O_2_A band (nm) under solar light
ΦPSII: photochemical yield of photosystem II, where the subscript FLD and PAM mean the value estimated by the FLD method and that measured by JUNIOR PAM, respectively
λ: wavelength
Chl: chlorophyll
DN: digital number
F band: Chl fluorescence intensity at O_2_A or O_2_B band (nm), where the subscript a and s in F mean artificial-light-induced and solar-induced values, respectively
FLD: Fraunhofer line depth principle
FWHM: full width at half maximum
LIFT: laser induced fluorescence transients
M: the mean values of radiant intensity (digital number), where the subscript a, b and c, d mean the bands of border and bottom of well from non-fluorescent reference and leaf, respectively
O_2_A: oxygen absorption band of 760 nm
O_2_B: oxygen absorption band of 686 nm
PAM: pulse-amplitude modulated
PPF: photosynthetic photon flux (μmol m^–2^ s^–1^)
PRI: photochemical reflectance index
R: reference coefficient
R^2^: coefficient of determination
RMSE: root mean square error
SIF: solar-induced Chl fluorescence
W: wavelength width (nm) at O_2_A or O_2_B band shown in Fig.1, the optimum is 0.4 nm.
